# Design and Fabrication of Membranes Based on PAN Copolymer Obtained from Solutions in *N*-methylmorpholine-*N*-oxide

**DOI:** 10.3390/polym14142861

**Published:** 2022-07-14

**Authors:** Igor S. Makarov, Markel I. Vinogradov, Lyudmila K. Golova, Natalia A. Arkharova, Gulbarshin K. Shambilova, Valentina E. Makhatova, Meirbek Zh. Naukenov

**Affiliations:** 1 A.V. Topchiev Institute of Petrochemical Synthesis, Russian Academy of Sciences, 29 Leninsky Prosp., 119991 Moscow, Russia; m.i.vinogradov1989@yandex.ru (M.I.V.); glk@ips.ac.ru (L.K.G.); 2 Federal Research Center “Crystallography and Photonics”, Russian Academy of Sciences, A.V. Shubnikov Institute of Crystallography, 59/1 Leninsky Prospekt, 119333 Moscow, Russia; natalya.arkharova@yandex.ru; 3 Department of Chemistry and Chemical Technology, Kh. Dosmukhamedov Atyrau University, Atyrau 060011, Kazakhstan; shambilova_gulba@mail.ru (G.K.S.); mahve@mail.ru (V.E.M.); 4 LLP «Atyrau Refinery», Atyrau 060001, Kazakhstan; meirbekk@mail.ru

**Keywords:** PAN, *N*-methylmorpholine-*N*-oxide, membranes, morphology, permeability, transport properties

## Abstract

An original method is proposed for preparing highly concentrated solutions of PAN copolymer in *N*-methylmorpholine-*N*-oxide (NMMO) and forming membranes for nanofiltration from these solutions. The high activity of the solvent with respect to the polymer provides short preparation time of spinning solutions in comparison with PAN solutions obtained in other solvents. The use of the rheological approach made it possible to find the optimal concentration for obtaining membranes. The formation of PAN membranes from the obtained solutions is proposed by the rolling method. The morphology of the formed membranes depends on the method of removing the precipitant from the sample. The features of the formed morphology of PAN membranes were studied by scanning electron microscopy. It was revealed that the use of water as a rigid precipitant leads to the formation of a homogeneous and symmetric morphology in the membrane. The average pore sizes in the membrane have been obtained by porosimetry. The study of the separating properties of PAN membranes revealed noteworthy values of the permeability and rejection for the anionic dyes Orange II and Remazol Brilliant Blue (74 and 97%, respectively). The mechanical properties of PAN membranes from solutions in NMMO are not inferior to analogs formed from commercially used direct solvents.

## 1. Introduction

Perhaps the intelligent use of organic membranes started in the beginning of the 19th century, when the first formulations of mechanisms explaining the processes occurring during the separation of liquid and gaseous media appeared [1]. After that, polymer membranes found new areas of application, and today they are used as packaging materials, for water purification, dialysis, in lithium batteries, optoelectronics, emulsion separation in the oil industry, etc. [2,3,4]. New technological solutions make it possible to improve the quality and productivity of the membranes obtained, to reduce their cost, and to increase mass application [5]. Depending on the obtained structure, polymer membranes can be divided into anisotropic and symmetric, porous and dense, with a different number of selective layers, etc. [6].

The range of polymers used for the production of membranes, over time, shifted from biopolymers and their derivatives to synthetic polymers. In recent decades, due to the increasing environmental requirements, there has been a reverse transition to natural polymers [7,8] and systems using nontoxic solvents [9]. In the case of synthetic polymers, polyacrylonitrile and its copolymers (PANs) are currently attracting great attention [10,11]. The structure and chemical structure of PAN limit the number of potential direct solvents and provide one of the main properties of future membranes from these polymers, namely, high chemical resistance [12,13,14]. Additional exposure of PAN membranes at temperature or exposure to IR radiation leads to cyclization processes in the polymer and an increase in resistance to traditional PAN solvents [15,16,17,18].

The most important directions of using PAN membranes such as desalination [19], microfiltration [20], ultrafiltration [21], are determined by the conditions of their preparation and the formed structure. The main method of preparing PAN membranes is their formation from solutions with subsequent removal of the solvent with a precipitator (phase inversion method) [22,23]. The formation of the structure of future membranes occurs at the moment of immersion of the membrane in the precipitator. The characteristics of the porous structure and of the entire membrane are mainly determined by the composition of the spinning solution (dope), the precipitation bath, and the conditions of the precipitation process [24]. For the first time, the phase inversion method of obtaining membranes was described in the 1960s of the last century in [25]. Upon contact of the precipitant with a thermodynamically equilibrium polymer solution, the system transforms from liquid to solid (non-fluid), with the formation of a polymer-rich phase and a phase with a low polymer content [26].

The decomposition of the system into phases upon contact of the solution with the precipitant during formation can proceed in two main directions [27]:

- Instant decomposition into phases occurs immediately after contact of the solution with the precipitant and leads to the formation of a porous upper layer and further elongated vacuoles;

- If, during the precipitation of a just formed sample in a precipitator, the replacement of the solvent occurs slowly and the rate of decomposition into phases is low, a dense morphology (possibly with a small amount of macropores) is formed in the upper layer, passing into a network structure.

Coagulation of the polymer is accompanied by the formation of a network structure at the microlevel; then, the removal of the liquid phase leads to the collapse of the voids, which, in turn, does not exclude the formed interphase boundaries [28]. In the future, these boundaries may play the role of hidden defects along which the membrane will be destroyed. Although the processes described above proceed at a high speed, they can be regulated, for example, by choosing a precipitant [29] or the temperature of the coagulation bath [30]. Thus, in [31,32] it was shown that an increase in the content of the solvent (DMSO) in the precipitation water bath allows to obtain fewer defective samples with a uniform cross-section. A decrease in temperature leads to a slowdown in diffusion processes and affects both the morphology and the shape of the resulting fibers [33]. Changing the chemical composition of the precipitation bath in the process of obtaining PAN membranes (films, fibers) is rarely used due to the technological difficulties in the regeneration of the precipitant and solvent. However, replacing water precipitation baths, for example, with methanol, makes it possible to obtain PAN fibers with a strength that is 40% higher in comparison with samples precipitated into water [34].

Another way to regulate membrane morphology is to increase the polymer concentration in solution [28]. An increase of concentration of the polymer in the system affects the coagulation rate, namely, decreases the diffusion rates of the precipitant and the solvent [35].

An increase of the PAN content in solution is difficult for widely used polar solvents (DMSO, DMF, DMAc, ionic liquids, ZnCl_2_, NaSCN, etc.) [36,37,38,39,40]. The polymer content in dopes based on these solvents is usually 12–18%, depending on the molecular weight of the polymer used. With a further increase of the PAN content in the system, the viscosity of the solutions increases significantly, exceeding the value of 10^2^ Pa * s. The increase in the viscosity of the solutions leads to various problems in the formation of membranes. We must not forget about the difficulties of working with these solvents, namely, the issues of regeneration, toxicity, high corrosiveness, etc. [39,40].

Further searches for new direct PAN solvents showed that the use of *N*-methylmorpholine-*N*-oxide (NMMO) makes it possible to obtain solutions with a polymer content of up to 55% [41]. As with the earlier mentioned solvents, the viscosity of such concentrated solutions is high. Therefore, the optimal concentration range of dopes varies from 20 to 40%. Without stopping on the mechanisms of PAN dissolution [42,43], we note that NMMO has been used on an industrial scale for many years [44], and its regeneration reaches 99.5% [45].

An increase of the concentration of PAN in the dope should undoubtedly affect the transport properties of membranes. Which leads to the question: Is it possible to use highly concentrated solutions of PAN in NMMO to obtain membranes for nanofiltration?

The objective of this research was to obtain membranes for nanofiltration based on PAN from highly concentrated solutions in NMMO, to study their structure and morphology, and to evaluate transport and mechanical properties.

## 2. Experimental

### 2.1. Materials

To obtain 28% solutions in *N*-methylmorpholine-*N*-oxide (NMMO), a ternary copolymer of PAN was used with the following composition: 93.9% acrylonitrile, 5.8% methyl acrylate, 0.3% sulfonic acid methyl ester (Mw = 85,000 g/mol) with an average particle size of 50 μm (Goodfellow, Huntingdon, UK). NMMO with Tm = 120 °C (water content~10%) manufactured by Demochem (Shanghai, China) was used as a PAN solvent. Thermooxidative destruction was inhibited by introducing 0.5% propyl gallate (Sigma-Aldrich, St. Louis, MI, USA).

### 2.2. Preparation of Dopes

The dopes were obtained by the method of solid-phase activation of the polymer–solvent system according to the procedures [41,46]. After activation, the solid-phase system was heated to the solvent melting temperature of 120 °C, was subjected to active mixing in the working unit of the HAAKE Minilab II twin-screw mixer (ThermoFisher Scientific, Dreieich, Germany), the rotation speed of the screws was 100 rpm. The preparation time of the dope did not exceed 2–5 min.

### 2.3. Rheology

The viscosity of PAN solutions was determined on a rotational rheometer HAAKE MARS 60 Rheometer (ThermoFisher Scientific, Dreieich, Germany) (the cone-plane measurement geometry, diameter 20 mm and angle 1°) under conditions of continuous deformation in the range of shear rates from 10^−3^ to 10^3^ s^−1^. To exclude drying of the sample, the sensor system was filled with PMS-100 silicone oil (OOO Silan, Moscow, Russia). The measurements were carried out at temperatures of 100–130 °C. The quality of PAN solutions in NMMO was assessed by polarizing optical microscopy (Boetius microscope (VEB Kombinat Nadema, formerly GDR)).

### 2.4. Preparation of the Membranes

The membranes were formed by rolling on a ChemInstruments HLCL-1000 laminator (ChemInstruments, Hamilton, OH, USA) at a temperature of 120 °C. To obtain a membrane of the required thickness, the solution was passed between the shafts of the laminator through a gap set using calibration plates. To exclude the adhesion of the PAN solution in NMMO to the rolls, two cover films made of polyimide and polyethylene terephthalate were used. To remove the solvent, after the formation of a thin layer of the solution, the top cover film was removed and the just formed system was immersed in a 1 L precipitation bath. Water at room temperature was used as a precipitant. The second substrate was removed after immersing of the sample in the bath. Upon contact with water, the solvent was removed from the film as a result of mass transfer processes, and the polymer was completely coagulated. The membrane was kept in a precipitation bath for 1 day. In order to exclude residual solvent from the membrane after coagulation, the samples were placed in water washing baths of the same temperature. The sample was kept in washing baths for a day. At the end of the day, the liquid in the bath was replaced with clean water. The procedure was repeated twice. After complete removal of the solvent, the membrane was not dried and was used to assess the mechanical and transport properties.

### 2.5. Mechanical Properties of Wet Membranes

The mechanical properties of the wet membranes were evaluated on an Instron 1122 tensile testing machine (Instron, Norwood, MA, USA) (stretching rate of 10 mm/min, a base between pneumatic clamps of 10 mm). Samples for mechanical testing were cut using a 4 × 1 cm sharp-edged frame. The tests were carried out at a temperature T = 22 ± 1 °C.

### 2.6. Morphology of PAN Membranes

The morphology of transverse cleavages of the dried membranes was investigated by low-voltage scanning electron microscopy (LVSEM) on an FEI Scios microscope (USA) at an accelerating voltage of less than 1 kV in the secondary electron mode [47]. For LVREM studies, the original membranes were dried by various methods: air drying, freeze drying, and chemical dehydration. In the latter case, drying was carried out with preliminary replacement of the nonsolvent with acetone according to the protocol [48]. Cleavages were made after freezing in liquid nitrogen perpendicular to the plane of the sample.

### 2.7. Structure of PAN Membranes

The structure of the initial PAN powder and the formed membranes was studied by X-ray diffractometry on a Rigaku Rotaflex D/MAX-RC setup equipped with a rotating copper anode (X-ray source operating mode 30 kV, 100 mA, characteristic radiation wavelength λ = 0.1542 nm, CuKβ radiation absorbed by a nickel filter), a horizontal goniometer, and a scintillation detector. X-ray experiments are performed in the reflection mode according to the Bragg–Brentano scheme in the continuous θ–2θ scanning mode in the angular range of 2.5–50.0° and a scanning step of 0.04° at room temperature.

### 2.8. Transport Properties

To assess the transport properties of PAN membranes, ethanol was used with a water content of 4 wt%. Rejection was estimated based on two anionic dyes Orange II (Sigma Aldrich, Taufkirchen, Germany) and Remazol Brilliant Blue R (Sigma Aldrich, Germany) with molecular weights of 350 and 626 g/mol, respectively. The Orange II (Remazol Brilliant Blue R) dye solutions in ethanol at a concentration of 30 mg/L were performed.

To determine the nanofiltration characteristics of membranes, dead-end cells (transmembrane pressure 20 atm.) were used according to the method described in [49]. The working area of the membrane in the cell was 16.6 cm^2^. The volume of liquid poured into the cell was 800 mL. The pressure in the cell was created using helium.

The permeate flow was determined by the gravimetric method. For this purpose, a liquid receiver was installed at the outlet of the cell, designed in such a way as to minimize the evaporation of the permeate (mainly the solvent) during the accumulation of the liquid sample. The measurement of the mass of permeate passing through the membrane during the experiment was carried out on a laboratory balance of the firm “Sartorius” (Germany) with a measurement error of 0.001 g. The membrane performance was characterized by the permeance of the liquid (*P*), which was calculated as follows:

(1) $$P=\frac{m}{S\;\cdot \;\Delta t\cdot \Delta p}$$

where *m* is the mass of permeate (kg) passed through a membrane with an area of *S* (m^2^) over a period of Δ*t* (h) and Δ*p* is the pressure drop.

The rejection **R** (%) was used to assess the separation properties of the membrane:

(2) $$R=(1-\frac{c_{P}}{c_{0}})\;\cdot \;100\%,$$

where $c_{0}$ and $c_{P}$ are concentrations of a dissolved substance in the initial testing solution and in the permeate, respectively.

The pore size distribution in the membranes was determined on a POROLIQ 1000 mL instrument (POROMETER, Vlaanderen, Belgium). The principle of operation of the device is based on the displacement of a non-wetting liquid. Wetting and non-wetting liquids were saturated solutions of water in isobutanol and isobutanol in water, respectively. The interfacial surface tension in the system used is 2 dyne/cm. Before starting the measurement, the sample was soaked for 24 h in ethanol to replace the water present in the pores, after which it was soaked in water-saturated isobutanol for 24 h. To determine the pore size, samples with a diameter of 2.5 cm were cut from the membrane, which were placed in isobutanol saturated with water for at least 24 h. The use of isobutanol saturated with water made it possible to minimize the effect of interdiffusion of two liquids at the initial moment of measurement. The sample soaked in isobutanol was placed into the cell, after which the measurement was started, during which the transmembrane pressure was increased stepwise from 0 until the characteristic S-shaped curve appeared on the graph of the flow versus the applied pressure, indicating the opening of all the pores of the membrane. At each pressure step, the measurement was carried out until the flow stabilized at the same level with an accuracy of ±1 μL/min for 30 s, or ±100 μL/min for flows greater than 1 mL/min.

## 3. Results and Discussion

**Figure 1:**
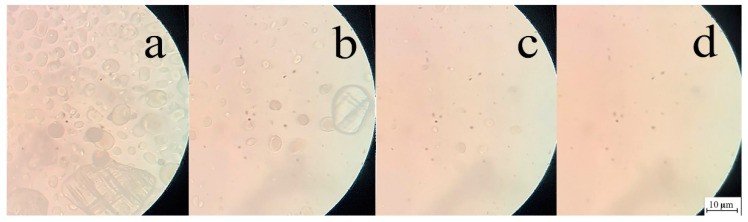
Evolution of dissolution of PAN in NMMO at 120 °C. Time from the beginning of the experiment: (**a**) 12; (**b**) 30; (**c**) 60; (**d**) 90 s.

The preparation of PAN solutions in NMMO made it possible to reduce the preparation time of solutions by several times in comparison with traditional solvents. To visualize the dissolution process, a solid-phase system containing 28% PAN powder in a crystalline solvent was placed between a slide and a cover glass and heated to 120 °C (Figure 1).

With the onset of heating, the crystals of the solvent melt and pass into a fluid state. Already 12 s after the start of the experiment, an active change in the particle sizes of the solvent and PAN powder is observed. After 30 s, single crystals of solvent and a small amount of swollen polymer particles are observed in the transmitted light of the microscope. Observing the evolution of PAN dissolution after a minute revealed minor traces of undissolved polymer. A completely isotropic field (i.e., dissolution of PAN in NMMO) was observed after 90 s. after the start of the experiment. Thus, under stationary conditions, 1.5 min was sufficient to obtain a homogeneous PAN solution.

**Figure 2:**
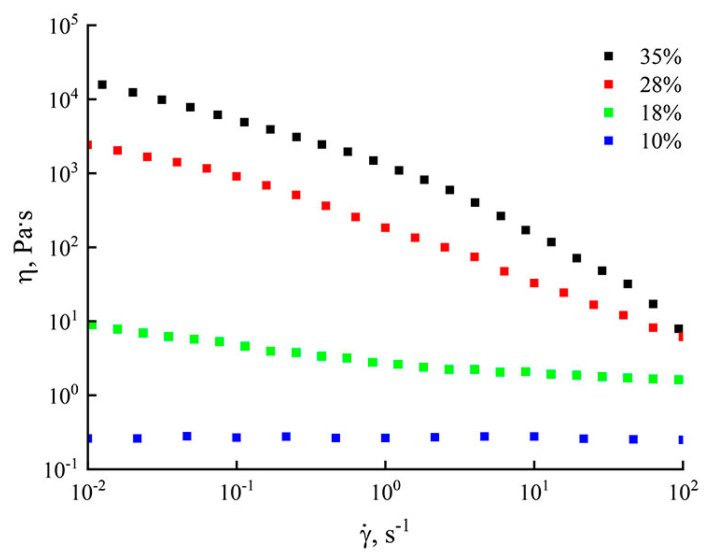
Flow curves of PAN solutions in NMMO at 120 °C.

The optimally high polymer concentration in the spinning solution in this study was selected according to the following characteristics: system preparation time, solution viscosity and formability, etc. It was shown above that a 28% PAN solution in NMMO can be prepared in 2 min. It is important to note that under the conditions of shear deformations and active mixing of the system, the time for preparing the solution can be reduced. The flow curves for the obtained PAN solutions of various concentrations in NMMO are shown in Figure 2.

The viscosity of solutions plays one of the key roles in the process of obtaining membranes. By varying the concentration of the polymer in the system, it is possible to change the viscosity of the solution by several times. Thus, the presented dependences showed that the transition from 10% solutions to 35% was accompanied by an increase in viscosity values by 5 orders of magnitude. For systems with a low content (up to 10%), a Newtonian flow was observed on the flow curves over the entire range of shear rates. Moving on to more concentrated solutions, in particular, with a content of 28% PAN, the region of the Newtonian flow was not observed, and the anomaly increased with an increase in the concentration of PAN. At speeds of more than 10^−2^ s^−1^, a decrease in viscosity was observed on the flow curve with an increase in the rate of deformation, which corresponded to the destruction of the structure of the solution. Systems with a polymer content of 35% similarly demonstrated a non-Newtonian flow pattern over the entire shear rate range.

**Figure 3:**
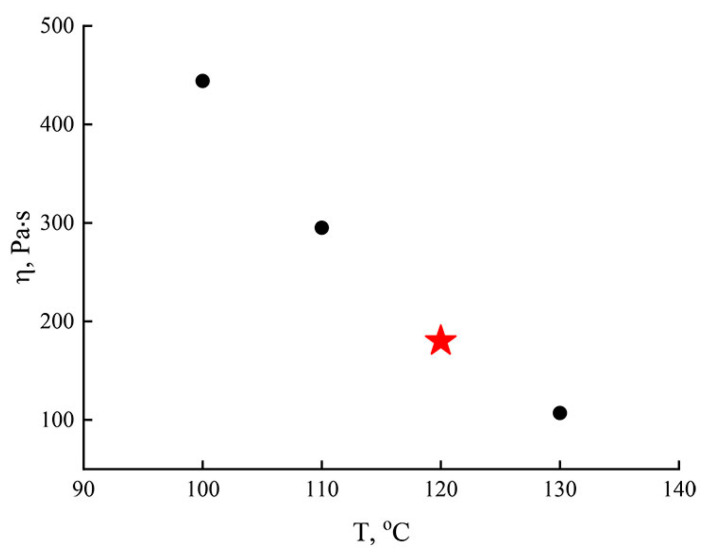
Temperature dependence of viscosity for a 28% PAN solution in NMMO (γ = 1 s^−1^).

Comparison of the rheological behavior of concentrated PAN solutions, as well as the time of their preparation, made it possible to identify the most appropriate polymer concentration in the system—28%. The temperature dependence of the viscosity for a given solution is shown in Figure 3.

Since the formation of films using extruders, rollers, etc., occurs at high shear rates from 1 s^–1^ and higher [50], the change in the viscosity of the solution with increasing temperature from 100 °C to 130 °C was considered at the corresponding rates. Figure 3 shows that an increase in temperature leads to a decrease in viscosity, which is traditional for PAN solutions. With an increase in temperature by 30 °C, the viscosity of the solution decreases four times and almost reaches 100 Pa*s (T = 130 °C). Analyzing the temperature dependence of the viscosity, taking into account the solution preparation temperature, from a practical point of view, the optimal membrane formation temperature was 120 °C (the point is marked with a star on the graph). The viscosity of the solution obtained at a given temperature is about 200 Pa*s, and with an increase in the shear rate to γ = 10^3^ s^−1^, its values decrease by two orders of magnitude. Thus, the obtained solutions with a polymer content of 28% were completely suitable for the formation of membranes, for example, by rolling.

**Figure 4:**
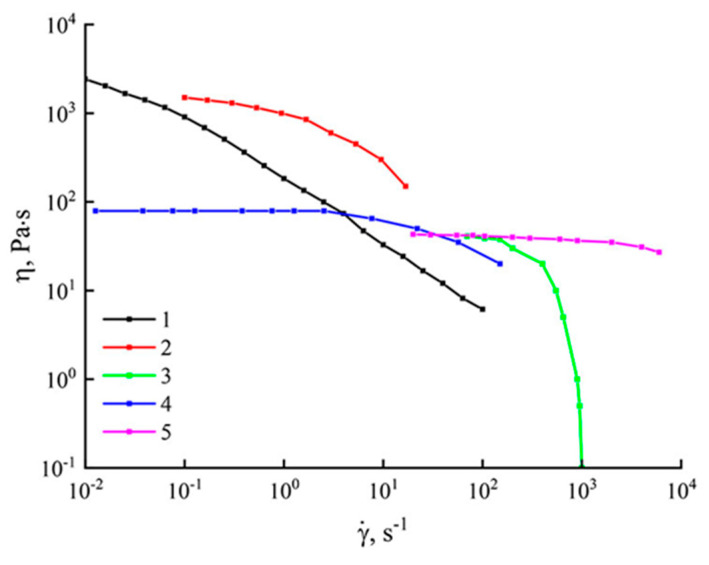
Flow curves of PAN solutions in various solvents (1–28% solution in NMMO (current work); 2–25% solution in DMSO at 30 °C [51]: 3–20% solution in [BMIN]Cl at 70 °C [52]; 4–11% solution in NaSCN at 25 °C [53]; 5–10% solution in DMF [54].

As mentioned in the theoretical part [36,37,38,39,40], the number of direct PAN solvents was limited. The concentration of dopes obtained using these solvents rarely exceeded 20%. Figure 4 shows the comparative flow curves for PAN solutions in various direct solvents [51,52,53,54].

The character of the flow curves for PAN solutions in DMF or, for example, in NaSCN containing up to 11% polymer coincides with equiconcentrated systems with NMMO as a solvent. For more concentrated solutions, the flow curves did not show a region of Newtonian behavior. An increase in the shear rate for these solutions led to a decrease in their viscosity. This was most likely due to the structural transformations in solutions.

Practice shows that if it is necessary to increase the polymer content in the system; an increase in the working temperature of the solution preparation is required. An increase in temperature reduces the viscosity of solutions and shortens the preparation time. For 25% systems in DMSO, solutions and flow curves for them were obtained at 30 °C, and in ionic liquids, 20% solutions were obtained at 70 °C. Due to the high cost of ionic liquids and the difficulties of their regeneration, these solvents are not widely used for the preparation of PAN membranes. Therefore, it makes no sense to dwell on such solutions in detail. Of greatest interest are solutions in the traditional PAN solvent DMSO and the new NMMO. The viscosity anomaly for these solutions increases with increasing shear rate. The viscosity of PAN solutions in NMMO (at γ = 1 s^−1^) is one order of magnitude lower compared to systems in DMSO. And although the rheological behavior of PAN solutions in NMMO and DMSO is similar, the time of obtaining solutions differs enormously in favor of the amine oxide. Thus, NMMO has a number of advantages over other direct solvents and can be used to form PAN membranes with minimal preparation times for concentrated solutions.

**Figure 5:**
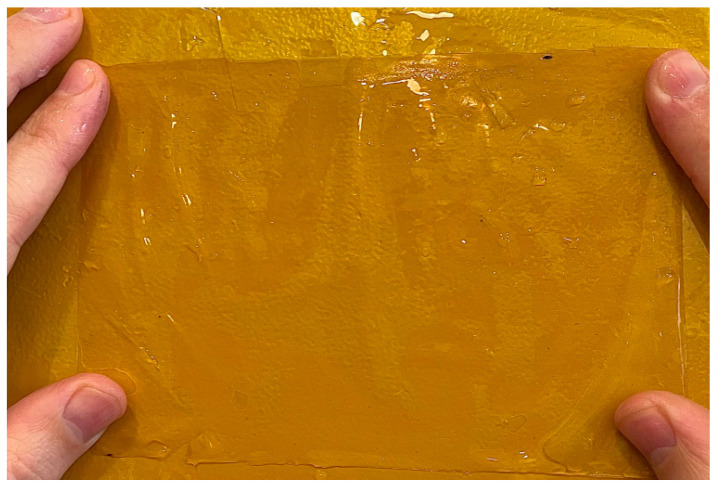
Photograph of a just formed PAN membrane after precipitation and washing with water.

PAN membranes were formed by passing the forming solution through the rollers of the laminator at a temperature of 120 °C. Figure 5 shows a sample of the membrane after removing the solvent.

As seen in the photo, the semitransparent PAN membrane was homogeneous, and there are no obvious defects.

The process of polymer coagulation during the preparation of polymer membranes from dopes is very often key. It is during this process that the structural features of the future membrane are laid. The use of “rigid” precipitators to remove the solvent leads to rapid mass transfer processes with the formation of large pores [54,55,56,57]. On the contrary, the use of “soft” precipitators allows the process of solvent removal to be carried out smoothly. The forming pores during such precipitation have a small scatter in size; large (finger-like) pores are absent [26,27,58]. Another way of forming a membrane in which large pores will not form is the use of concentrated dopes [59].

**Figure 6:**
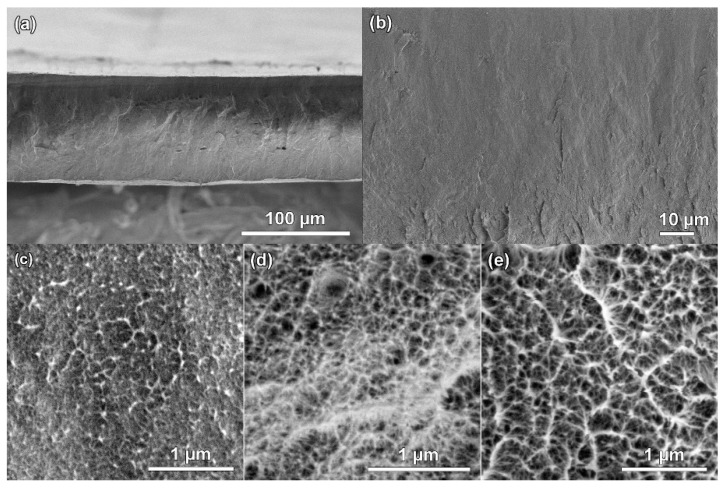
SEM images of transverse cleavages of PAN membranes: (**a**–**c**) dried at room conditions, (**d**) freeze drying; (**e**) replacement of a liquid medium and drying.

SEM images of the surface morphology of transverse cleavages of membranes obtained from 28% PAN solutions in NMMO are shown in Figure 6.

It is known that sample preparation for SEM studies is fundamental for obtaining reliable information on the structure of initial polymeric materials containing water or other organic liquids. To optimize the method of sample preparation of membranes based on PAN for SEM studies, various drying techniques were used. Note that there are practically no differences in the structure at the macrolevel for membranes depending on the drying conditions. Figure 6a–c show SEM images of the membrane cleavage after drying in air at room temperature. It can be seen that the microstructure of the transverse cleavage of the membrane is symmetric, monolithic (homogeneous), and practically defect free. Exceptions are rare areas of air accumulation, which disappear with the subsequent degassing of the dope. Despite the fact that the edges of the film dried in air were even smooth, and defects caused by drying the sample did not appear on the surface, it was not possible to obtain information on the pore size in the sample (Figure 6c). SEM images of the morphology of the membrane cleavage dried using the universal method—freeze-drying and using the chemical dehydration method according to the standard protocol, where acetone was used as a substitute nonsolvent, as shown in Figure 6d,e, respectively. It is clear that the membrane has a cellular structure with a network of interpenetrating pores. From SEM images of membrane cleavages dried by the last two methods, it was determined that the pore sizes vary from a few nanometers to 100 nm. In this case, the cell size is up to 500 nm. The data obtained made it possible to gain an idea of the transformation of the morphology of the membrane based on PAN when using various drying methods, as well as to optimize the sample preparation for SEM studies.

Thus, it was shown that the morphology observed using scanning microscopy strongly depends on the method of drying the sample. To refine the above data on pore sizes by SEM, additional methods are required, for example, porosimetry, in which a non-wetting liquid is displaced, and the results obtained make it possible to determine the average pore size for PAN-based membranes.

Here, one can draw a kind of analogy between drying samples with preliminary substitution of a non-solvent (water-> ethanol-> acetone) and preparing a sample for porosimetry, where the membrane was soaked for 24 h in ethanol before the experiment, and then in isobutanol saturated with water. As a result, the average pore size was determined, which is in the range of 2–5 nm, which is a small pore size for membranes obtained from PAN. In this case, the permeability of aqueous solutions of isobutanol through them is in the range of 1.66–3.65 L/(m^2^ h atm). The order of the obtained permeability values for PAN membranes from solutions in NMMO is close to the published data on the permeability of nanofiltration membranes. Thus, in works [60,61] for ethanol the permeability was ~4.2 (L * m ^−2^ * h ^−1^ * bar ^−1^), and for isopropanol 1.9 (L * m ^−2^ * h ^−1^ * bar ^−1^) [62,63]. In the case of PAN, from which ultrafiltration membranes are usually obtained, their permeability values (water) are one to two orders of magnitude higher and vary from 40 to 6150, depending on the method of membrane formation [18,64]. The revealed morphology and data on the pores of the samples obtained in this work allow us to consider them as nanofiltration membranes.

The separating properties of the obtained membranes were evaluated by the permeability of ethanol and its solutions with dyes, the Rejection coefficients were studied for the anionic dyes Remazol Brilliant Blue R and Orange II (Table 1). For all systems, the values were estimated after the end of the period of initial relaxation of the membrane and reaching the steady-state flow regime.

From the values presented in the table, it can be seen that in the case of an alcoholic solution of Remazol Brilliant Blue R, the maximum values of the rejection coefficient are achieved. For Orange-based systems, observed values are 20% lower. Thus, we can speak of a good selectivity of PAN membranes formed from highly concentrated solutions in NMMO.

The resulting polymer membranes are often subjected to mechanical stress during their operation. In addition, in the case of unsatisfactory strength properties, destruction of the membrane is possible with a complete loss of transport and separation properties. Proceeding from this, it is reasonable to begin the study of the properties of a new type of membranes precisely from the study of their mechanical behavior.

PAN films often lose their elasticity during the drying process and can become brittle. This is due to the fact that during the drying of the membrane, a radical change in structure and morphology is possible. Small pores can permanently collapse, large pores, defect areas, etc., are formed [65].

On the other hand, in some cases, dry polymer membranes are preconditioned in a liquid prior to use. Hence, it is of interest to study the mechanical characteristics of membranes in the wet state, i.e., not dried after removing the solvent with water. The obtained values of strength, modulus of elasticity and elongation at break are presented in Table 2.

The revealed mechanical properties, although inferior to the values obtained for dry membranes [60], allow considering these samples to study their transport properties. The difference in mechanical properties for samples cut along and across the molding axis is practically not manifested. The independence of the mechanical characteristics from the direction for the obtained films is associated with the peculiarities of the formed structure in the membrane.

**Figure 7:**
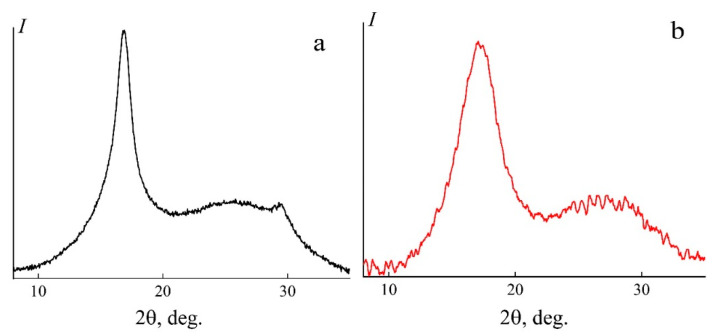
Diffraction patterns of the initial powder (**a**) and film (**b**) of the PAN terpolymer (AN / MA / MS). Scanning mode: reflection.

Figure 7 shows diffraction patterns for a dry membrane and the initial powder of the ternary copolymer PAN (AN/MA/MS).

Typical diffraction patterns for PAN contain two main reflections: the first maximum with a higher intensity is located in the region of the diffraction angle 2θ~17° (100). The second less intense peak is in the region of the diffraction angle 2θ~29° (110). The second reflection is characterized by the superposition of an additional amorphous peak on it, with a maximum in the region 2θ = 25–27°. The structural picture of the terpolymer powder used in this work fully corresponds to the classical diffraction patterns of PAN [24,66,67]. The diffraction pattern (Figure 7a) contains two distinct peaks at 2θ = 16.9° (d = 0.524 nm) and 2θ = 29.4° (d = 0.303 nm), as well as a wide peak at 25.7°. In the case of a PAN membrane, the intensity of peak 1 at 2θ = 16.9° (d = 0.528 nm) decreases, while its half-width increases. Peak 2 (2θ~29°) is completely overlapped by the amorphous component of the diffractogram 2θ~25–29°. The displayed diffraction pattern for PAN membranes, according to [68,69], corresponds to an ordered crystalline phase with a layered structure and an amorphous phase with a disordered arrangement of chains.

## 4. Conclusions

Thus, for the first time, a method was proposed for preparing membranes for nanofiltration from highly concentrated solutions of PAN in NMMO. In contrast to the previously described methods for preparing PAN membranes, the one presented in this work allows one to obtain highly concentrated spinning solutions in short time intervals. The use of the rolling method made it possible to form PAN membranes of unlimited geometry (the variable width is determined by the dimensions of the rollers). The mechanical properties of the formed membranes were at a level corresponding to this type of membrane and are not inferior to those already described in the literature. The surface morphology and cleavage structure of PAN membranes were studied by scanning electron microscopy. It was shown that the precipitation of 28% PAN solutions in NMMO with water leads to the formation of a homogeneous finely spongy morphology in contrast to polymer solutions in DMSO, DMF, etc. The average size of the observed pores varies from 2 to 5 nanometers. Revealed high selectivity of PAN membranes in relation to Remazol dye, which reached 97%. Further work on the selection of precipitation conditions for formed membranes (composition and temperature of precipitation baths) will open up possibilities for regulating their morphology and transport properties. The proposed method for obtaining concentrated solutions can be used to form hollow fiber membranes (PAN hollow fiber supported TFC membrane) for use in forward osmosis.

## Figures and Tables

**Table 1:** Ethanol permeance, Orange II and Remazol Brilliant Blue R dye rejection for PAN membranes.

Sample	P_EtOH_, kg/m^2^ h bar	P_EtOH+Remazol_, kg/m^2^ h bar	R_Remazol_, %	P_EtOH+Orange_, kg/m^2^ h bar	R_Orange_, %
PAN membrane	0.6	0.58	97	0.6	74

P—Permeance; R—rejection.

**Table 2:** Mechanical properties of wet PAN membranes formed in water media and washed with water.

Sample	Tensile Strength (MPa)	Young’s Modulus (GPa)	Elongation at Break (%)
PAN membrane	16 ± 2	0.32 ± 0.07	38 ± 5

## Data Availability

Not applicable.
